# Predictive models and treatment efficacy for liver cancer patients with bone metastases: A comprehensive analysis of prognostic factors and nomogram development

**DOI:** 10.1016/j.heliyon.2024.e38038

**Published:** 2024-09-19

**Authors:** Peng Qiu, Yunxiang Feng, Kai Zhao, Yuanxin Shi, Xiangyu Li, Zhengdong Deng, Jianming Wang

**Affiliations:** a Department of Biliary and Pancreatic Surgery, Cancer Research Center Affiliated Tongji Hospital, Tongji Medical College, Huazhong University of Science and Technology, Wuhan, China; b Department of Pediatric Surgery, Tongji Hospital of Tongji Medical College, Huazhong University of Science and Technology, Wuhan, China; c Affiliated Tianyou Hospital, Wuhan University of Science & Technology, Wuhan, China

**Keywords:** Primary liver cancer, Bone metastasis, SEER, Prognosis, Nomogram, Risk factors, PSM, Surgery, Radiotherapy, Chemotherapy

## Abstract

**Background:**

Bone metastasis considerably undermines the prognosis of advanced primary liver cancer patients. Though its impact is well-recognized, the clinical field still lacks robust predictive models that can accurately forecast patient outcomes and aid in treatment effectiveness evaluation. Addressing this gap is paramount for improving patient management and survival.

**Materials and methods:**

We conducted an extensive analysis using data from the SEER database (2010–2020). COX regression analysis was applied to identify prognostic factors for primary liver cancer with bone metastasis (PLCBM). Nomograms were developed and validated to predict survival outcomes in PLCBM patients. Additionally, propensity score matching and Kaplan-Meier survival analyses lent additional insight by dissecting the survival advantage conferred by various treatment strategies.

**Results:**

A total of 470 patients with PLCBM were included in our study. The median overall survival (OS) and cancer-specific survival (CSS) for these patients were both 5 months. We unveiled several independent prognosticators for OS and CSS, spanning demographic to therapeutic parameters like marital status, cancer grade, histological type, and treatments received. This discovery enabled the formulation of two novel nomograms—now verified to eclipse the predictive prowess of the traditional TNM staging system regarding discrimination and clinical utility. Additionally, propensity score matching analysis showed the effectiveness of surgeries, radiotherapy, and chemotherapy in improving OS and CSS outcomes for PLCBM patients.

**Conclusions:**

Our investigation stands out by introducing pioneering nomograms for prognostic evaluation in PLCBM, a leap forward compared to existing tools. Far exceeding mere academic exercise, these nomograms hold immense clinical value, serving as a foundation for nuanced risk stratification systems and delivering dynamic, interactive guides, allowing healthcare professionals and patients to assess individual bone metastasis survival probabilities and personalize treatment selection.

**Table:** Abbreviations

PLCBM	Primary liver cancer with bone metastasis
OS	Overall survival
CSS	Cancer-specific survival
HCC	Hepatocellular carcinoma
ICC	Intrahepatic cholangiocarcinoma
PSM	Propensity score matching
ROC	The receiver operating characteristics curves
AUC	DCA The area under the curve Decision curve analysis

## 1 Introduction

Primary liver tumors, including hepatocellular carcinoma (HCC) and intrahepatic cholangiocarcinoma (ICC), rank as the sixth highest prevalence and the third major contributor to cancer-related fatalities on a global scale. These aggressive malignancies contribute to an estimated 900,000 new cases and approximately 830,000 fatalities each year [1]. While the combination of radical surgery and non-operative treatments like radiation therapy, chemotherapy, targeted therapy, and immunotherapy has substantially enhanced the outlook for early-stage liver cancer patients, those with advanced liver cancer, especially with distant metastasis, face limited treatment options and poor outcomes. Bone metastasis, a frequent complication in advanced liver cancer, trailing only pulmonary metastasis, has notable implications on patient survival and morbidity [2]. Empirical evidence from prior studies has consistently demonstrated that the presence of bone metastasis holds substantial prognostic significance for patients with advanced liver cancer [3].

Despite the detrimental consequences of bone metastasis in liver cancer, it has historically received limited attention, possibly due to its relatively lower prevalence in previous decades. Consequently, patients with bone metastasis experience a significant deterioration in their quality of life and an alarmingly low median overall survival of less than 6 months [[4], [5], [6]]. This pessimistic prognosis can be attributed, at least in part, to the lack of curative treatment options for bone metastasis, with current approaches like chemotherapy, radiotherapy, and surgical resection being primarily palliative [3,[7], [8], [9]]. Furthermore, the prognosis of patients with bone metastasis in primary liver cancer is strongly influenced by various clinical characteristics [10].

To address these challenges and meet the needs of patients in terms of prognosis, it is crucial to develop precise prognostic prediction models that accurately estimate survival in individuals with advanced liver cancer and bone metastasis. Previous studies have largely focused on hepatocellular carcinoma and ignored intrahepatic cholangiocarcinoma, probably because of its low incidence. However, recent data demonstrate that bone metastasis is not uncommon in advanced stages of ICC, especially when distant metastasis is present [11,12]. This oversight necessitates a reevaluation of existing data, especially since studies focusing on HCC with bone metastasis have operated on limited case numbers, potentially undermining the reliability of past conclusions. Our study, therefore, seeks to bridge this knowledge gap by exploring prognostic factors influencing the survival of patients with bone metastasis stemming from primary liver cancers including both HCC and ICC. By leveraging recent data from the comprehensive SEER (Surveillance, Epidemiology, and End Results) database, we aspire to construct accurate prognostic nomograms for both overall survival (OS) and cancer-specific survival (CSS) that predict survival rates at key intervals of 6, 12, and 18 months. Additionally, we propose to evaluate the prognostic impact of three prevalent treatment approaches using propensity score matching (PSM), providing valuable insight into the most efficacious treatment options for this patient demographic.

## 2 Materials and methods

### 2.1 Data source and variable definitions

To ensure the reliability and comprehensiveness of our study, we utilized the SEER database by SEER∗Stat software version 8.4.1, which is known for its extensive coverage and authority. This database collects tumor-related information from approximately 30 % of the entire population of the United States, providing us with a substantial sample size of newly diagnosed patients with primary liver cancer accompanied by bone metastasis from 2010 to 2020. To establish consistent criteria for participant selection, we applied specific inclusion and exclusion criteria. The inclusion criteria were as follows: (1) patients with a histological diagnosis confirming the presence of liver cancer with bone metastasis, and (2) patients diagnosed between 2010 and 2020. Conversely, we excluded patients who had missing information regarding primary site, metastatic site, demographic characteristics, and histopathological data. Additionally, individuals with incomplete survival and follow-up data were also excluded from the study. Patients enrolled in the study were monitored and followed up until one of the following events occurred: death, loss to follow-up, or the predefined cut-off date of December 31, 2020. The collected demographic and clinical information in this study included patient ID, age, race, marital status, year of diagnosis, tumor grade, histological subtype, T stage, N stage, tumor dimensions, presence of brain and lung metastases, surgical interventions, radiotherapy, chemotherapy, cause of death classification, and survival data. To facilitate further analysis, continuous variables such as tumor size were grouped. Patients were categorized into two groups according to the age at which they were diagnosed ( ≤ 60 versus >60). In the marital status category, the "Others" group included widowed, separated, and divorced individuals. The surgical options for PLCBM patients were classified into two categories: “no surgery” or “surgery,” according to the SEER Program Coding and Staging Manual. The main outcomes of the study were overall survival (OS) and cancer-specific survival (CSS). Overall survival (OS) was defined as the duration from the initial diagnosis to the date of the last follow-up, considering all-cause mortality. This means that any cause of death, regardless of whether it was related to PLCBM or not, was taken into account in calculating OS. Cancer-specific survival (CSS) focused specifically on the time period from the initial diagnosis until the date of the last follow-up, but only considering deaths directly attributed to PLCBM. CSS provided a measure of survival specifically related to PLCBM and accounted for deaths caused by the disease itself.

### 2.2 Nomogram Construction and validation

In this study, a random allocation method was used to divide the patient cohort into training and validation cohorts. The ratio of this allocation was 7:3, with the majority of patients assigned to the training cohort. This division ensured that a sufficient number of cases were available for model development while maintaining an independent dataset for validation purposes.

In the training cohort, univariate and multivariate Cox proportional hazard regression models were employed to identify independent prognostic factors for PLCBM patients. Univariate analysis allowed for the examination of individual factors, while multivariate analysis accounted for the simultaneous effects of multiple factors and identified those that were independently associated with prognosis. Nomograms were constructed based on the results of the Cox regression models. These nomograms served as predictive tools for estimating the 6-, 12-, and 18-month overall survival (OS) and cancer-specific survival (CSS) probabilities for PLCBM patients. Nomograms provide a visual representation of the prognostic model and facilitate individualized survival predictions.

To evaluate the accuracy of the training and validation cohorts, time-dependent receiver operating characteristics (ROC) curves and the area under the curve (AUC) were utilized. These metrics assess the model's discriminatory ability to distinguish between different survival outcomes. Calibration curves were also used to assess the model's overall performance and its calibration accuracy in predicting survival probabilities. Additionally, decision curve analysis (DCA) was employed to further validate the performance of the predictive model. DCA is a statistical method commonly used to evaluate the clinical utility and benefit of a predictive model by measuring the net benefit across a range of threshold probabilities.

### 2.3 Statistical analysis

In the study, continuous variables were reported using medians and interquartile ranges (IQR), which provide a summary measure of the data's central tendency and variability. Categorical variables were presented as integers and percentages, indicating the frequency and proportion of patients falling into each category. To compare non-normally distributed continuous variables between different groups, the Mann-Whitney *U* test was used. This non-parametric test assesses whether there are statistically significant differences in the distributions of the variables. For comparisons between categorical variables, the chi-square test or Fisher's exact test was employed. The chi-square test is used when comparing categorical variables with more than two categories, while Fisher's exact test is used when comparing categorical variables with a small sample size. To investigate the impact of three treatment modalities on the prognosis of PLCBM patients, a propensity score matching (PSM) method was employed. Propensity scores were calculated using a multifactorial Cox analysis, and patients were matched in a 1:1 ratio based on statistically significant variables obtained from the analysis. Propensity score matching helps to reduce the bias and confounding effects of treatment assignment. The prognosis of patients was assessed using Kaplan-Meier curves, which provide a visual representation of survival probabilities over time. These curves allow for the comparison of different treatment modalities and can provide insights into the potential differences in prognosis. Statistical analyses were conducted using SPSS 24.0 software and R software (version 4.2.1). SPSS is a widely used statistical software package, while R is a popular programming language and software environment for statistical computing and graphics. The two-tailed P-value was used to determine statistical significance, with a threshold of less than 0.05 indicating significance.

## 3 Results

### 3.1 Clinical features and survival outcomes in patients with PLCBM

**Fig. 1:**
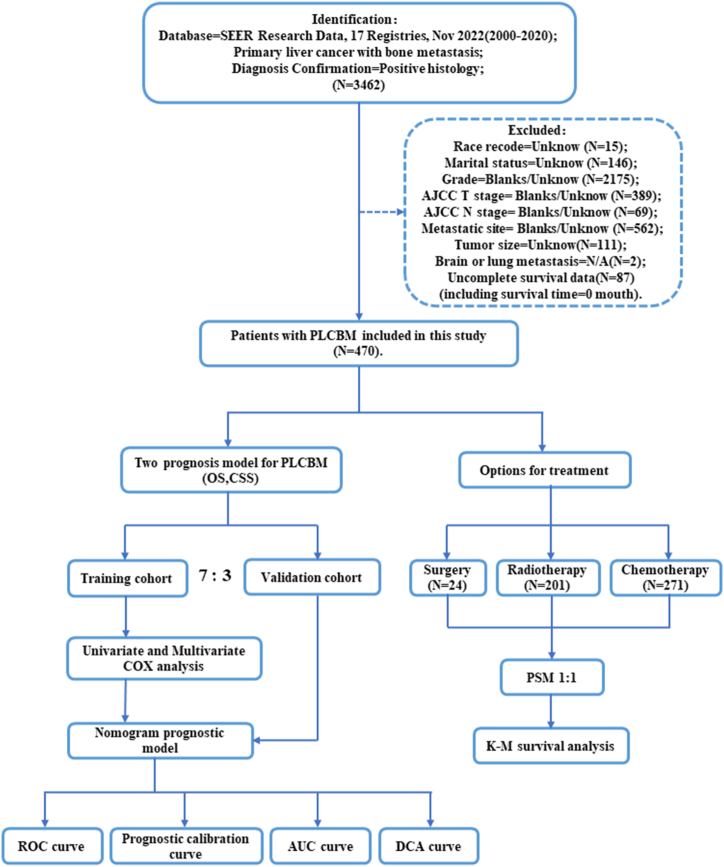
Flow chart of this study.

**Fig. 2:**
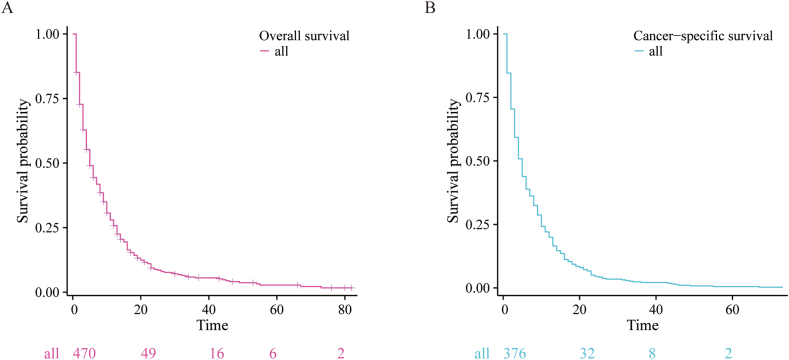
The KM curve of overall survival (OS) and cancer-specific survival (CSS) in all patients with primary liver cancer with bone metastasis (PLCBM).

In this retrospective study, a cohort of patients diagnosed with primary liver cancer with bone metastasis (PLCBM) between 2010 and 2020 was selected from the Surveillance, Epidemiology, and End Results (SEER) database. The patient selection process is visually illustrated in Fig. 1, outlining the inclusion and exclusion criteria. A total of 470 patients meeting these criteria were included for analysis. Notably, out of these patients, 376 experienced mortalities attributed to PLCBM, thus enabling us to investigate cancer-specific survival outcomes. Of all the PLCBM patients, 71.5 % were over 60 years old, with the majority being male (80.7 %), white (71.9 %), and married (56.6 %). The most common grade stages identified were Grade II (39.2 %) and Grade III (37.7 %), while Grade I (21.9 %) and Grade IV (1.2 %) were less common. Hepatocellular carcinoma was the most common histological type observed. In terms of AJCC T stage, T1 (31.3 %) and T3 (37.4 %) were the most commonly identified stages, while T2 (22.8 %) and T4 (8.5 %) were less frequently observed. Regarding lymph node metastasis, 70.9 % of the patients had N0 status, with 29.1 % having N1 status. With regards to extraosseous metastasis, lung metastasis (25.3 %) was the most frequent site, while brain metastasis (3.6 %) was the least common. The majority of patients underwent chemotherapy (57.6 %), while nearly half of the patients underwent radiotherapy (42.8 %). A smaller percentage of patients underwent surgery (8.8 %). It is worth noting that some patients may have received a combination of these treatments for additional management of their PLCBM. Utilizing the Kaplan-Meier methodology, we further examined the prognostic factors in patients with PLCBM and the comprehensive results obtained from this analysis are graphically represented in Fig. 2. Patients diagnosed with PLCBM had a median overall survival (OS) of 5 months. The 6-months survival rate was 44.35 %, which decreased to 25.75 % at 12 months and 14.24 % at 18 months. In terms of cancer-specific survival (CSS), patients with PLCBM had a median CSS of 5 months. The 6-months survival rate for CSS was 38.83 %, which decreased to 19.95 % at 12 months and 9.31 % at 18 months.

### 3.2 Identifying prognostic factors in patients with PLCBM

To ensure the reliability and impartiality of our findings, a randomized assignment process was employed, allocating all patients into two distinct cohorts - the training cohort and the validation cohort - in a ratio of 7:3. Table 1 comprehensively presents the complete dataset, encompassing demographic information and clinicopathological characteristics of the patients. Importantly, there were no obvious disparities observed between the training and validation groups, indicating a well-balanced distribution suitable for training and validating our model. In the training cohort, both univariable and multivariable Cox regression analyses were conducted to identify prognostic factors that influenced overall survival (OS) and cancer-specific survival (CSS) in patients diagnosed with PLCBM. The results of the univariable Cox regression analysis are summarized in Table 2, while variables with a p-value of less than 0.15 were subsequently included in the multivariable analysis to ensure the inclusion of potentially relevant factors. From the multivariable analysis, we discovered that marital status, grade, histological type, surgery, radiotherapy, and chemotherapy emerged as independent risk factors for overall survival. Similarly, grade, histological type, lung metastasis, surgery, radiotherapy, and chemotherapy were identified as independent risk factors for cancer-specific survival. It is worth noting that patients with single marital status exhibited significantly worse overall survival, while the presence of lung metastasis was associated with reduced cancer-specific survival. Notably, patients with hepatocellular carcinoma (HCC) subtype demonstrated improved overall survival and cancer-specific survival compared to those with cholangiocarcinoma (CCA). Additionally, surgery, radiotherapy, and chemotherapy were determined to be effective treatment modalities, resulting in improved overall survival and cancer-specific survival outcomes.

**Table 1:** Clinical characteristics of primary liver cancer patients with bone metastasis in training and validation cohorts.

Characteristics	All	Training cohort	Validation cohort	P value
n	470	329	141	
Age, n (%)				0.789
≤60	134 (28.5 %)	95 (20.2 %)	39 (8.3 %)	
>60	336 (71.5 %)	234 (49.8 %)	102 (21.7 %)	
Race, n (%)				0.401
White	333 (71.9 %)	227 (48.3 %)	106 (22.6 %)	
Black	66 (14.0 %)	49 (10.4 %)	17 (3.6 %)	
Other	71 (15.1 %)	53 (11.3 %)	18 (3.8 %)	
Marital status, n (%)				0.997
Married	266 (56.6 %)	186 (39.6 %)	80 (17 %)	
single	113 (19.3 %)	64 (13.6 %)	27 (5.7 %)	
Other	91 (24.1 %)	79 (16.8 %)	34 (7.2 %)	
Sex, n (%)				0.741
Male	379 (80.7 %)	264 (56.2 %)	115 (24.5 %)	
Female	91 (19.3 %)	65 (13.8 %)	26 (5.5 %)	
Grade, n (%)				0.380
Well differentiated; Grade I	103 (21.9 %)	70 (14.9 %)	33 (7.0 %)	
Moderately differentiated; Grade II	177 (39.2 %)	125 (26.6 %)	59 (12.6 %)	
Poorly differentiated; Grade III	184 (37.7 %)	131 (27.9 %)	46 (9.8 %)	
Undifferentiated; anaplastic; Grade IV	6 (1.2 %)	3 (0.6 %)	3 (0.6 %)	
Histological type, n (%)				0.340
HCC	353 (75.1 %)	243 (51.7 %)	110 (23.4 %)	
CCA	117 (24.9 %)	86 (18.3 %)	31 (6.6 %)	
AJCC T stage, n (%)				0.657
T1	147 (31.3 %)	99 (21.1 %)	48 (10.2 %)	
T2	107 (22.8 %)	78 (16.6 %)	29 (6.2 %)	
T3	176 (37.4 %)	126 (26.8 %)	50 (10.6 %)	
T4	40 (8.5 %)	26 (5.5 %)	14 (3.0 %)	
AJCC N stage, n (%)				0.808
N0	333 (70.9 %)	232 (49.4 %)	101 (21.5 %)	
N1	137 (29.1 %)	97 (20.6 %)	40 (8.5 %)	
Tumor size, median (IQR)	79 (55, 112)	78 (55, 114)	80 (56, 110)	0.649
Brain metastasis, n (%)				0.258
No	453 (96.4 %)	315 (67.0 %)	138 (29.4 %)	
Yes	17 (3.6 %)	14 (3.0 %)	3 (0.6 %)	
Lung metastasis, n (%)				0.594
No	351 (74.7 %)	248 (52.8 %)	103 (21.9 %)	
Yes	119 (25.3 %)	81 (17.2 %)	38 (8.1 %)	
surgery, n (%)				0.715
No	446 (94.9 %)	313 (66.6 %)	133 (28.3 %)	
Yes	24 (5.1 %)	16 (3.4 %)	8 (1.7 %)	
Radiotherapy, n (%)				0.887
No	269 (57.2 %)	189 (40.2 %)	80 (17.0 %)	
Yes	201 (42.8 %)	140 (29.8 %)	61 (13.0 %)	
Chemotherapy, n (%)				0.076
No	271 (42.4 %)	148 (31.5 %)	51 (10.9 %)	
Yes	199 (57.6 %)	181 (38.5 %)	90 (19.1 %)

**Table 2:** Univariate and multivariate logistics analysis of overall survival (OS) and cancer-specific survival (CSS) in patients with PLCBM.

Characteristics	Univariate analysis				Multivariate analysis			
	OS		CSS		OS		CSS	
	Hazard ratio (95 % CI)	P value	Hazard ratio (95 % CI)	P value	Hazard ratio (95 % CI)	P value	Hazard ratio (95 % CI)	P value
Age		0.245		0.783				
≤60	Reference		Reference					
>60	0.861 (0.670–1.106)	0.240	1.037 (0.799–1.345)	0.784				
Race		0.710		0.284				
White	Reference		Reference					
Black	0.968 (0.700–1.339)	0.846	0.904 (0.643–1.272)	0.562				
Other	1.131 (0.825–1.551)	0.445	1.283 (0.902–1.823)	0.166				
Marital status		**0.034**		0.135				
Married	Reference		Reference		Reference		Reference	
Single	1.476 (1.108–1.967)	**0.008**	1.216 (0.902–1.640)	0.200	1.397 (1.042–1.874)	**0.026**	0.921 (0.672–1.261)	0.608
Other	1.106 (0.836–1.463)	0.482	1.350 (0.985–1.850)	0.062	0.924 (0.693–1.232)	0.592	1.295 (0.933–1.796)	0.122
Sex		0.665		0.668				
Male	Reference		Reference					
Female	0.940 (0.709–1.247)	0.667	1.074 (0.776–1.487)	0.666				
Grade		**0.011**		**0.014**				
Grade I	Reference		Reference		Reference		Reference	
Grade II	1.245 (0.911–1.700)	0.169	1.160 (0.834–1.613)	0.378	1.210 (0.880–1.665)	0.241	1.121 (0.796–1.579)	0.512
Grade III	1.656 (1.209–2.268)	**0.002**	1.529 (1.099–2.128)	**0.012**	1.732 (1.230–2.438)	**0.002**	1.426 (1.000–2.035)	**0.050**
Grade IV	0.942 (0.295–3.006)	0.919	16.304 (2.189–121.462)	**0.006**	1.481 (0.454–4.829)	0.515	7.890 (1.028–60.564)	**0.047**
Histological type		0.126		0.103				
HCC	Reference		Reference		Reference		Reference	
CCA	0.815 (0.625–1.064)	0.133	0.798 (0.606–1.051)	0.109	0.714 (0.524–0.973)	**.**	0.688 (0.503–0.942)	**0.019**
AJCC T stage		0.610		0.174				
T1	Reference		Reference					
T2	1.056 (0.780–1.429)	0.724	1.070 (0.769–1.489)	0.690				
T3	1.032 (0.782–1.361)	0.824	1.280 (0.948–1.727)	0.107				
T4	1.399 (0.870–2.248)	0.166	1.592 (0.980–2.587)	0.060				
AJCC N stage		0.548		0.464				
N0	Reference		Reference					
N1	1.080 (0.841–1.386)	0.546	1.108 (0.844–1.453)	0.460				
Tumor size	0.999 (0.997–1.002)	0.503	1.001 (0.999–1.003)	0.438				
Brain metastasis		0.380		0.330				
No	Reference		Reference					
Yes	0.786 (0.451–1.372)	0.397	1.337 (0.763–2.343)	0.310				
Lung metastasis		**0.048**		**0.010**				
No	Reference		Reference		Reference		Reference	
Yes	1.307 (1.008–1.693)	**0.043**	1.454 (1.104–1.915)	**0.008**	1.218 (0.935–1.587)	0.145	1.367 (1.025–1.823)	**0.033**
Surgery		**< 0.001**		**< 0.001**				
No	Reference		Reference		Reference		Reference	
Yes	0.451 (0.274–0.742)	**0.002**	0.411 (0.227–0.745)	**0.003**	0.313 (0.185–0.531)	**< 0.001**	0.230 (0.120–0.442)	**< 0.001**
Radiotherapy		**< 0.001**		**< 0.001**				
No	Reference		Reference		Reference		Reference	
Yes	0.665 (0.526–0.841)	**< 0.001**	0.648 (0.505–0.831)	**< 0.001**	0.720 (0.562–0.923)	**0.009**	0.674 (0.513–0.887)	**0.005**
Chemotherapy		**< 0.001**		**< 0.001**				
No	Reference		Reference		Reference		Reference	
Yes	0.538 (0.426–0.678)	**< 0.001**	0.493 (0.385–0.632)	**< 0.001**	0.559 (0.431–0.727)	**< 0.001**	0.494 (0.371–0.656)	**< 0.001**

### 3.3 Constructing and validating two prognostic nomograms for patients with PLCBM

**Fig. 3:**
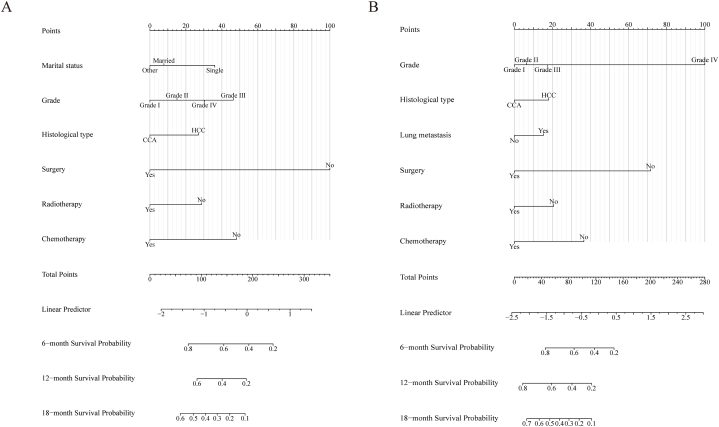
The nomograms for predicting the overall survival (OS) and cancer-specific survival (CSS) in patients with PLCBM.

**Fig. 4:**
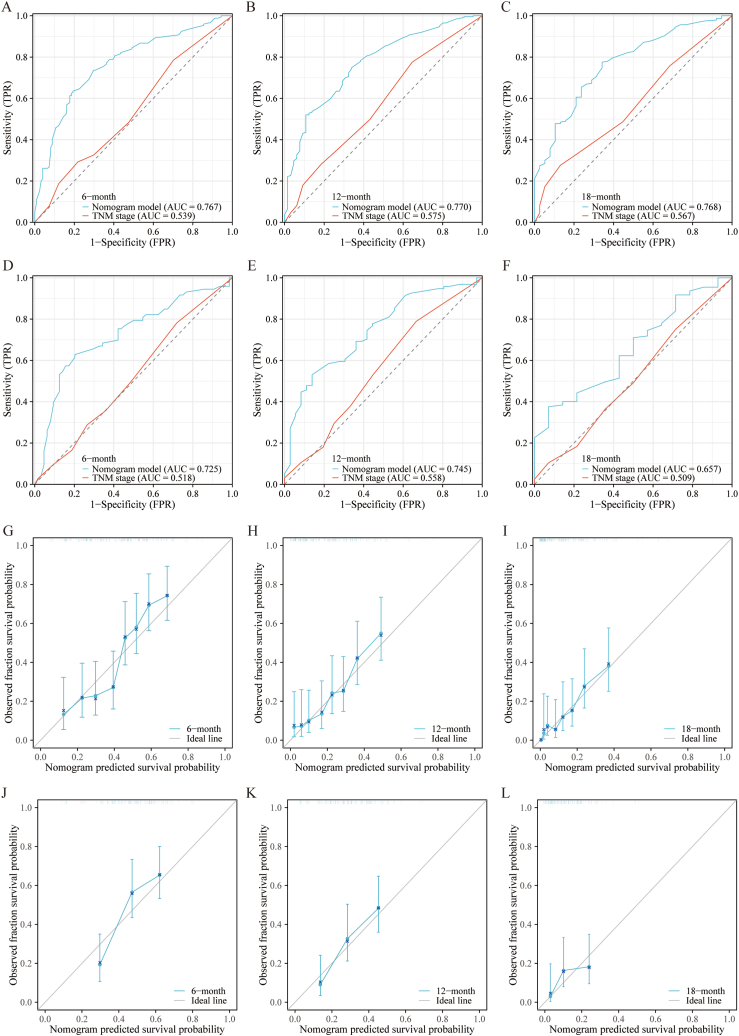
The receiver operating characteristic (ROC) curve and the calibration curves of the TNM stage and the overall survival (OS) nomogram in training and validation cohorts. (A–F) Time-dependent receiver operating characteristics (ROC) curves at 6-months (A), 12-months (B), and 18-months (C) in the training cohorts and at 6-months (D), 12-months (E), and 18-months (F) in the validation cohorts; (G–L) The calibration curves at 6 months (G), 12 months (H), and 18 months (I) in the training cohorts and at 6 months (J), 12 months (K), and 18 months (L) in the validation cohorts.

**Fig. 5:**
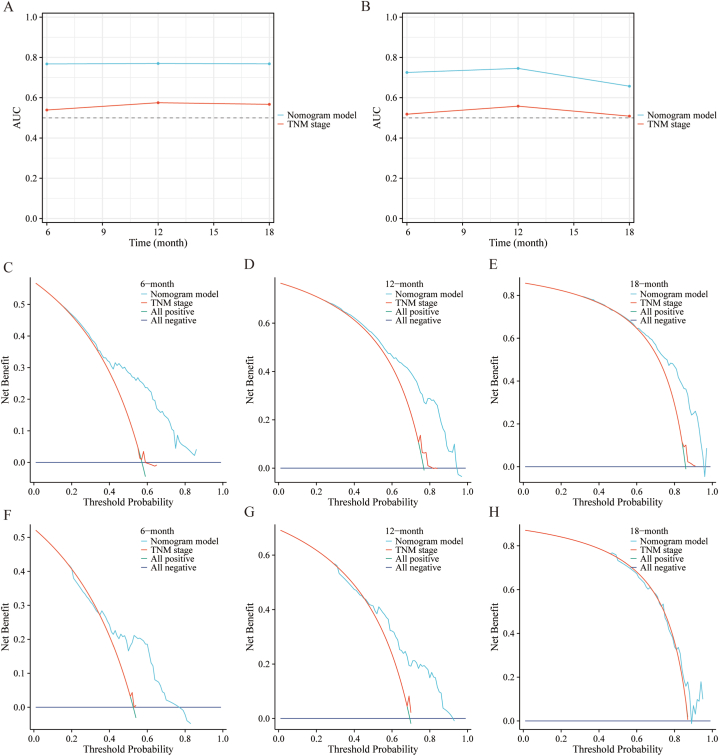
The area under the curve (AUC) and decision curve analysis (DCA)of the TNM stage and the overall survival (OS) nomogram. (A–B) The AUC predicting the overall survival (OS) between 6-months and 18-months in training cohorts (A) and validation cohorts (B); the DCA at 6-months (A), 12-months (B), and 18-months (C) in the training cohorts and at 6-months (D), 12-months (E), and 18-months (F) in the validation cohorts.

**Fig. 6:**
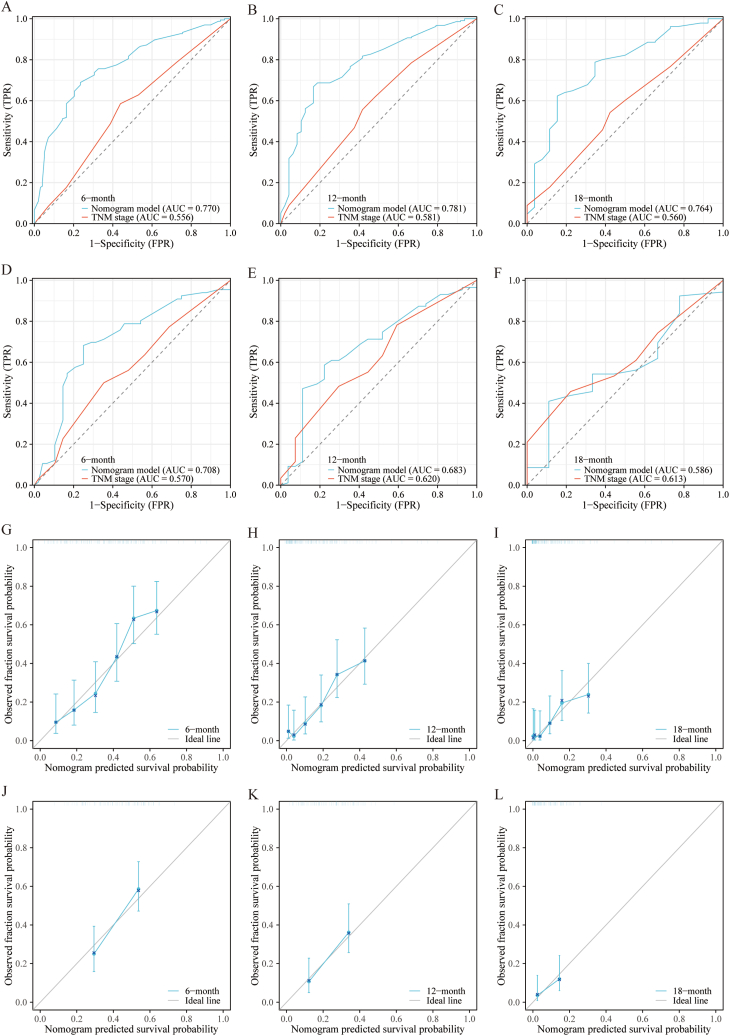
The receiver operating characteristic (ROC) curve and the calibration curves of the TNM stage and the cancer-specific survival (CSS) nomogram. (A–F) The ROC curves at 6-months (A), 12-months (B), and 18-months (C) in the training cohorts and at 6-months (D), 12-months (E), and 18-months (F) in the validation cohorts; (G–L) the calibration curves at 6 months (G), 12 months (H), and 18 months (I) in the training cohorts and at 6 months (J), 12 months (K), and 18 months (L) in the validation cohorts.

**Fig. 7:**
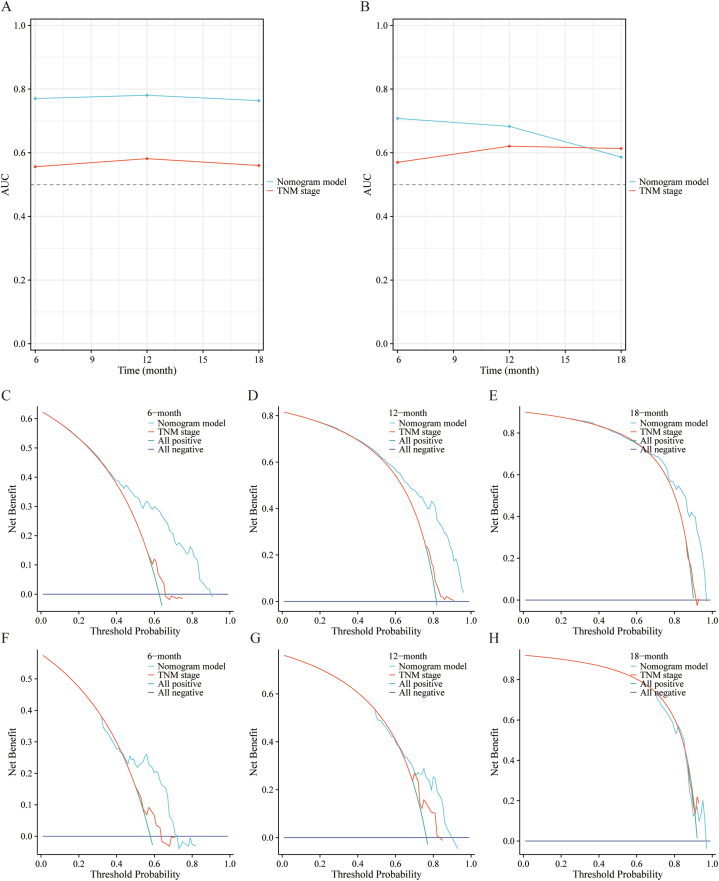
The area under the curve (AUC) and decision curve analysis (DCA)of the TNM stage and the cancer-specific survival (CSS) nomogram. (A–B) The AUC predicting the cancer-specific survival (CSS) between 6-months and 18-months in training cohorts (A) and validation cohorts (B); the DCA at 6-months (A), 12-months (B), and 18-months (C) in the training cohorts and at 6-months (D), 12-months (E), and 18-months (F) in the validation cohorts.

To illustrate the impact of the independent predictors for OS and CSS, we developed two predictive prognostic nomograms (Fig. 3). This calculator is constructed relying on the independent factors identified in our study and enables clinicians to assess the influence of these factors on both OS and CSS outcomes. To assess the performance of our predictive nomograms for PLCBM patients, we employed various evaluation methods including ROC curves, AUC curves, calibration curves, and decision curve analysis (DCA) (Fig. 4, Fig. 5, Fig. 6, Fig. 7). In terms of overall survival (OS), the nomogram demonstrated significantly higher AUC values compared to the TNM stage system. Specifically, the AUC values for predicting 6-months, 12-months, and 18-months overall survival using the nomograms were 0.767, 0.770, and 0.768, respectively, in the training cohort (Fig. 4A–C). The AUC values in the validation cohort for predicting the same time frames were 0.725, 0.745, and 0.657, respectively (Fig. 4D–F). Time-dependent AUC curves further illustrated that the AUC values remained relatively stable around 0.75 from 6 to 18 months in the training cohort, outperforming the TNM stage system (Fig. 5A). Interestingly, the range of AUC values in the validation cohort exhibited notable consistency with that observed in the training cohort (Fig. 5B). These results indicate that the OS nomogram outperforms the AJCC TNM stage system in prognostication. The similar pattern was observed in the nomogram predicting cancer-specific survival (CSS) where the AUC values were relatively higher compared to the traditional TNM stage system for predicting 6-months, 12-months, and 18-months cancer-specific survival in both the training and validation cohorts (Fig. 6A–F and Fig. 7A–B). Additionally, the calibration curves demonstrated that the predictions made by both models were consistent with the actual observations in both the training and validation cohorts, which suggested that the nomograms we developed are effective tools for accurately predicting OS and CSS in patients with PLCBM. Fig. 4G–L shows the calibration curves for the OS nomogram, while Fig. 6G–L displays the calibration curves for the CSS nomogram. In decision curve analysis (DCA), the nomogram curves consistently surpassed those of the TNM stage system in both prognostic models for OS and CSS, in both the training and validation groups（Fig. 5C–H and Fig. 7C–H）,which confirmed the accuracy of the prediction tool in forecasting the survival prognosis of patients with PLCBM. These findings highlight the substantial net gains and practical value in clinical settings offered by both the OS and CSS nomograms beyond the traditional TNM stage system.

### 3.4 Survival benefit of three treatment modalities for patients with PLCBM

**Fig. 8:**
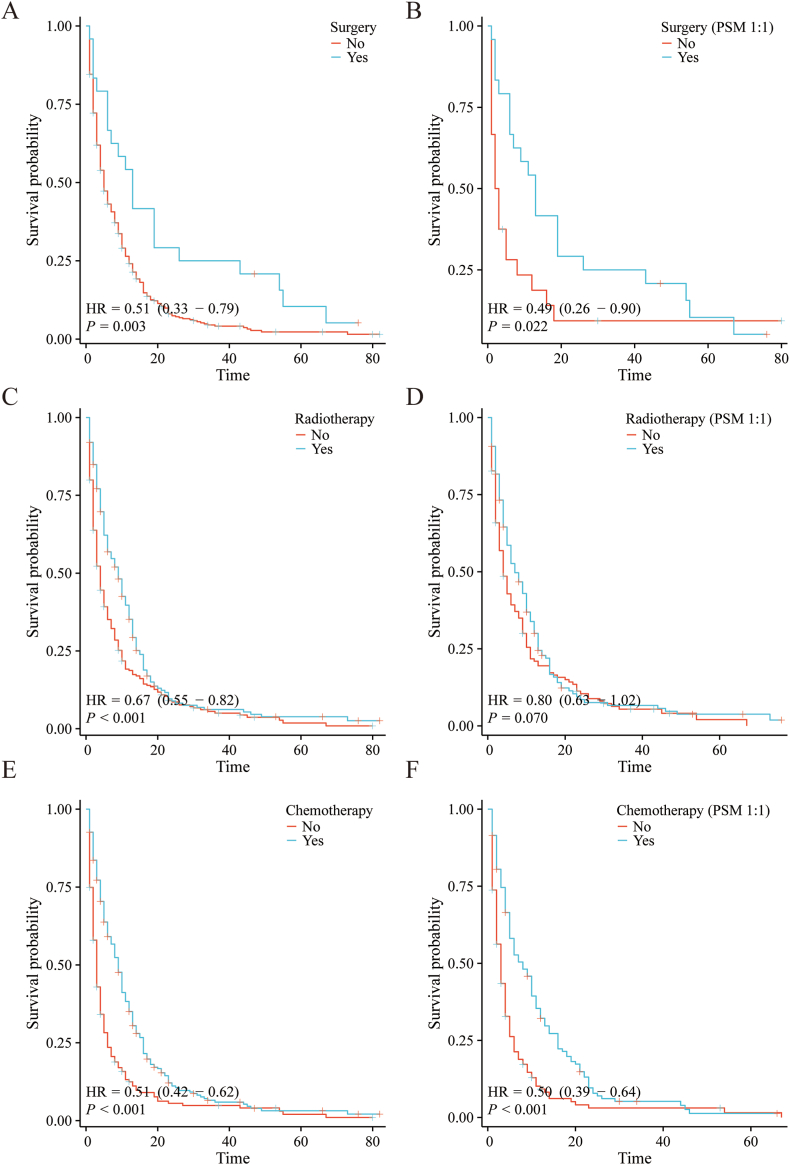
The overall survival (OS) of three treatment modalities for patients with PLCBM. (A–B) The overall survival (OS) of surgery for PLCBM before and after PSM; (C–D) The overall survival (OS) of radiotherapy for PLCBM before and after PSM; (E–F) The overall survival (OS) of chemotherapy for PLCBM before and after PSM.

**Fig. 9:**
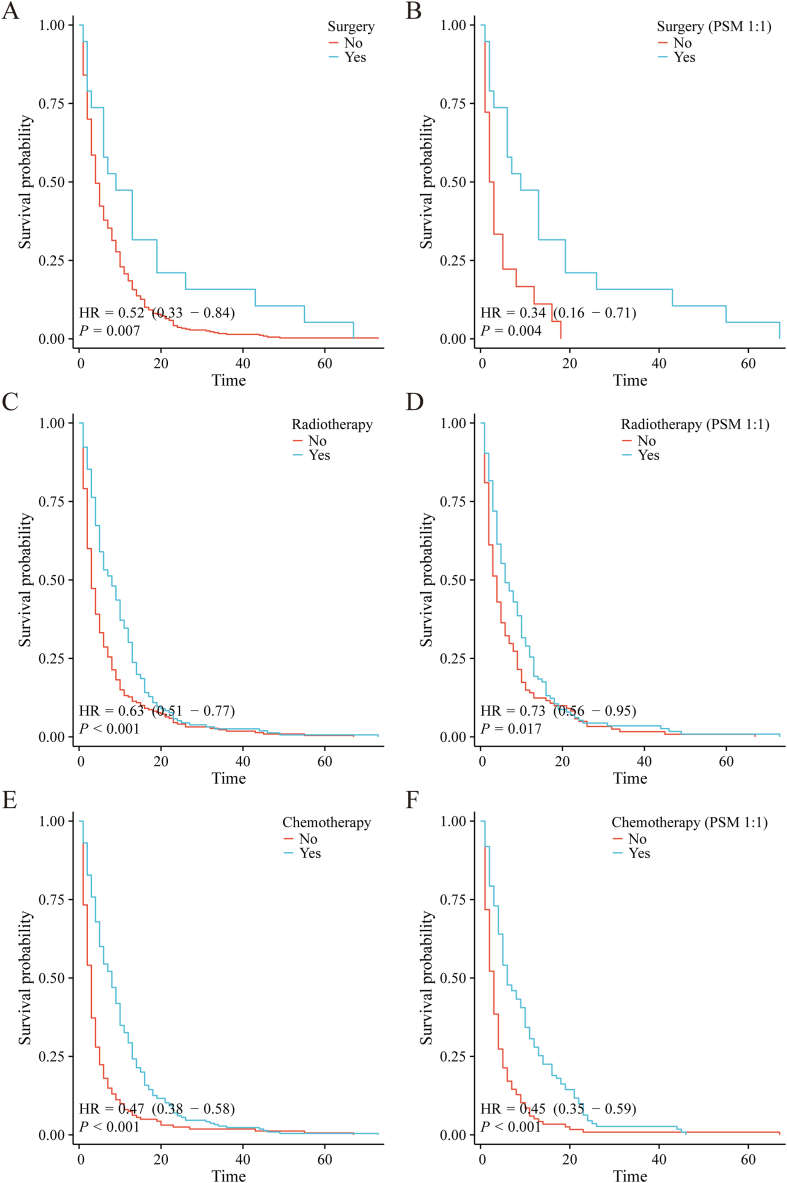
The cancer-specific survival (CSS) of three treatment modalities for patients with PLCBM. (A–B) The cancer-specific survival (CSS) of surgery for PLCBM before and after PSM; (C–D) The cancer-specific survival (CSS) of radiotherapy for PLCBM before and after PSM; (E–F) The cancer-specific survival (CSS) of chemotherapy for PLCBM before and after PSM.

Given the three treatment modalities as the major independent risk factors, we further investigated the role of three treatment ways in the prognosis of PLCBM. Initially, a comparison of baseline characteristics was performed between different treatment groups: surgery versus non-surgery, radiotherapy versus non-radiotherapy, and chemotherapy versus non-chemotherapy. These comparisons revealed notable differences in some clinical factors. To mitigate the impact of these biases and confounding variables, propensity score matching (PSM) was employed to adjust for the existing imbalances. Following PSM adjustment, there was no obvious differences noted in basic features between the compared groups, demonstrating improved compara bility (Table S1-3). K-M curves were performed to analyze the prognosis of patients in the different treatment groups. Prior to PSM adjustment, it was observed that patients who underwent surgery exhibited significantly better overall survival (OS) (Fig. 8A) (p = 0.003, HR: 0.51; 95 % CI: 0.33–0.79) and cancer-specific survival (CSS) (Fig. 9A) (p = 0.007, HR: 0.52; 95 % CI: 0.33–0.84) compared to those who did not undergo surgery. Likewise, patients who received radiotherapy or chemotherapy demonstrated significantly improved OS (Fig. 8C and E) and CSS (Fig. 9C and E) compared to non-recipients (radiotherapy: OS p < 0.001, HR: 0.67; 95 % CI: 0.55–0.82 and CSS p < 0.001, HR: 0.63; 95 % CI: 0.51–0.77; chemotherapy: OS p < 0.001, HR: 0.51; 95 % CI: 0.42–0.62 and CSS p < 0.001, HR: 0.47; 95 % CI: 0.38–0.58). Following PSM adjustment, it was observed that patients who underwent surgery maintained significantly better OS (Fig. 8B) (p = 0.022, HR: 0.49; 95 % CI: 0.26–0.90) and CSS (Fig. 9B) (p = 0.004, HR: 0.34; 95 % CI: 0.16–0.71) compared to those who did not receive surgery. Furthermore, patients who underwent radiotherapy had a relatively better OS (Fig. 8D) (p = 0.070, HR: 0.80; 95 % CI: 0.63–1.02) and a significantly better CSS (Fig. 9D) (p = 0.017, HR: 0.73; 95 % CI: 0.56–0.95) compared to their counterparts who did not receive radiotherapy, while patients who received chemotherapy demonstrated significantly better OS (Fig. 8F) (p < 0.001, HR: 0.50; 95 % CI: 0.39–0.64) and CSS (Fig. 9E) (p < 0.001, HR: 0.45; 95 % CI: 0.35–0.59) compared to those who did not receive chemotherapy. These observations indicate that surgery, radiotherapy, and chemotherapy are all effective treatment modalities associated with improved survival rates in the studied population.

## 4 Discussion

Bone metastasis is a pervasive occurrence in advanced hepatic malignancy, often indicative of a grave prognosis. About 29.4 % of cases involving metastasis entail the dissemination of malignant cells to skeletal tissues [2]. According to a study including 380 patients underwent hepatectomy, about 11.4 % patients developed bone metastasis during a median follow-up period of 54.3 months [13]. For patients with advanced liver cancer who have developed bone metastases, the focus shifts to estimating their survival time, as these cases are generally considered incurable. However, accurate prediction models for prognosis remain lacking in clinical settings. In our study, we aimed to address this gap by establishing and validating two prognostic nomograms for OS and CSS. Importantly, our research offers a thorough analysis that includes complete data on the clinical characteristics and prognosis of patients with advanced liver cancer and bone metastases up to the present date. The survival rates at 6, 12, and 18 months for patients with bone metastases from liver cancer were found to be 44.35 %, 25.75 %, and 14.24 % in terms of OS, respectively. Similarly, the CSS rates for the same timepoints were 38.83 %, 19.95 %, and 9.31 %, respectively. This study identified several independent prognostic factors for OS and CSS. Patients diagnosed with PLCBM who are single, have a poorly differentiated tumor grade, or suffer from hepatocellular carcinoma (HCC) face a significantly higher risk of decreased overall survival (OS). Additionally, the absence of treatments like surgical intervention, radiotherapy, and chemotherapy further exacerbates this risk. Similarly, patients with a poorly differentiated or undifferentiated tumor grade, a diagnosis of HCC, the presence of lung metastasis, or inadequate receipt of treatments exhibit a significantly decreased cancer-specific survival (CSS). These findings highlight the importance of considering these factors when assessing the prognosis of PLCBM. Based on the identified prognostic factors, we developed two nomograms for predicting overall survival (OS) and cancer-specific survival (CSS). Fig. 3 illustrates how patients were assigned scores for each parameter, and the cumulative scores determined the patient's 6-, 12-, and 18-month OS and CSS probabilities. The performance of these nomograms was validated using the area under the curve (AUC) and calibration curves, confirming their excellent accuracy and discriminatory capabilities. Notably, decision curve analysis (DCA) demonstrated that the nomograms possessed higher clinical value compared to the traditional TNM staging system. These findings highlight the precise and efficient predictive ability of the constructed nomograms in assessing patient survival. Firstly, the relationship between marital status and OS deserves attention. Single PLCBM patients have been found to face a significantly higher risk of decreased overall survival. This consequence was consistent with previous study [13]. This observation may be attributed to the potential lack of social support and caregiving assistance compared to married individuals [14]. Considering the influence of marital status on individuals well-being and healthcare decision-making, healthcare providers should consider providing additional support and resources to single PLCBM patients to potentially improve survival outcomes.

Moreover, tumor grade emerges as a significant prognostic factor for both OS and CSS. Poorly differentiated tumor grade is associated with worse survival outcomes in PLCBM patients. Tumor grade reflects the degree of cellular differentiation and aggressiveness. Patients with poorly differentiated or undifferentiated tumors often exhibit more rapid disease progression and a higher likelihood of treatment resistance. Therefore, close monitoring of tumor grade and timely intervention may be essential in managing PLCBM and improving survival rates.

One important prognostic factor identified in our study is the pathological type of hepatocellular carcinoma (HCC). Patients with PLCBM who suffer from a pathological type of HCC have a higher risk of decreased overall survival. Different pathological types of liver cancer may exhibit varying aggressiveness and response to treatment, thereby influencing patient outcomes. Intrahepatic Cholangiocarcinoma is generally regarded as a more aggressive tumor compared to hepatocellular carcinoma, with a generally worse prognosis [15]. But in our study, HCC with bone metastasis had a poorer prognosis comparing with ICC and the specific reason is unknown. Further research should explore the molecular and genetic characteristics of different liver cancer subtypes in the context of PLCBM to develop targeted therapies and improve survival rates.

Furthermore, the influence of different treatments on survival outcomes is worth emphasizing. The absence of treatments, such as surgical intervention, radiotherapy, and chemotherapy, significantly exacerbates the risk of decreased overall survival both before and after PSM. Similarly, inadequate receipt of treatments leads to lower cancer-specific survival rates. In the past, extrahepatic metastasis of liver cancer was often seen as a terminal stage of the disease, and surgical resection was not typically considered as a treatment option [16]. However, advancements in medical knowledge and surgical techniques have led to a shift in this perspective. Today, there is growing evidence to suggest that surgical resection may be beneficial for select patients with extrahepatic metastasis of liver cancer [2,[17], [18], [19]]. Surgical resection of extrahepatic metastases aims to remove the cancerous growths in other organs or lymph nodes while also treating the primary liver tumor. This approach can help alleviate symptoms, improve quality of life, and potentially extend survival in some cases. This is especially true in cases where the metastasis is limited and can be completely removed, and where the overall health of the patient is good. It is crucial to recognize that not all patients with extrahepatic metastasis of liver cancer will be eligible for surgical resection. The decision to pursue surgery will depend on various factors, such as the site and extent of the metastasis, the overall stage of the cancer, the patient's overall health, and their ability to tolerate the surgery and recuperate afterwards. The limited number of patients undergoing surgery for this condition may result in a lack of statistical power and reliability in the findings. To establish more reliable evidence and guidelines, it is important to conduct large-scale studies that involve a greater number of patients. Radiotherapy is a commonly used treatment for bone metastasis from liver cancer [9,[20], [21], [22]]. This treatment method utilizes high-energy radiation to eliminate cancer cells, alleviate pain and discomfort, and control or slow down the progression of bone metastasis. It not only has the potential to extend the survival time of patients but also to enhance their quality of life, as supported by our findings. In cases of advanced liver cancer, chemotherapy is often highly recommended as a treatment option. This recommendation also applies to patients who have developed bone metastasis [23]. Chemotherapy exerts its effects on both the primary liver tumor and metastatic lesions, including those in the bone. By targeting cancer cells in the bone, chemotherapy can help to alleviate bone pain, halt disease progression, and improve overall survival. Besides, the administration of bone-modifying agents is recommended in patients with bone metastasis from liver cancer [24,25]. Bisphosphonates, such as zoledronic acid and pamidronate, and the monoclonal antibody denosumab have been shown to have beneficial effects on bone health. These agents inhibit osteoclast activity, reducing bone resorption and the risk of fractures, as well as helping to alleviate bone pain. Moreover, they have the potential to prevent SREs, such as pathological fractures and spinal cord compression, which can significantly impact patients' quality of life. And our study similarly found chemotherapy could prolong the patient's survival time. These findings underscore the importance of early and appropriate treatment interventions to improve patient outcomes. Multidisciplinary treatment approaches involving oncologists, surgeons, radiologists, and supportive care teams should be considered to ensure the optimal management of PLCBM.

The strength of our research lies in the development and validation of predictive models for the overall survival (OS) prognosis and cancer-specific survival (CSS) prognosis in patients with bone metastasis from primary liver cancer (PLC). These predictive models, which outperform the traditional TNM stage system, are represented by nomograms and provide valuable insights for precise clinical decision-making and monitoring in individual patients. By inputting relevant data into the corresponding nomogram, clinicians can easily calculate a total score to accurately determine the prognostic risk for patients with PLC bone metastasis. Besides, all the information needed for analysis is complete, without any missing data. the completeness of our data enhances the reliability and validity of our findings. The absence of missing data reduces the risk of bias and strengthens the overall quality of our research. This allows for a more comprehensive analysis and increases the credibility of the predictive models developed in our study. Moreover, by incorporating PSM analysis into our study, we aimed to enhance the understanding of the prognosis of patients with bone metastasis from PLC who received different therapies. This methodological approach strengthens the validity and reliability of our findings, providing valuable insights for clinical decision-making and future research in this field.

However, it is important to acknowledge several limitations within our study. Firstly, the relatively small sample size of patients with PLC bone metastasis (PLCBM) may have introduced bias. Additionally, while machine learning, a branch of artificial intelligence that mimics human learning and improves through various flexible algorithms, offers computational efficiency, manages complex relationships, and enhances predictive accuracy, it is increasingly used in both medicine and non-medicine [[26], [27], [28]]. However, we did not employ machine learning in this study. Instead, we used the COX regression analysis model to identify prognostic factors and develop prognostic models. It is important to note that some machine learning models may lack interpretability and require substantial data. Future research should focus on larger sample sizes and the use of more advanced machine learning algorithms. Secondly, the lack of specific information on bone metastasis (BM) sites in the SEER database poses a limitation, as this information significantly impacts patient survival prognosis. It can be inferred that patients with multiple and extensive BM generally have shorter survival times. Thirdly, the retrospective nature of our study raises concerns about potential selection bias. Additionally, the SEER database's lack of detailed treatment information further limits the scope of our findings.

## 5 Conclusions

In summary, our population-based study, using the SEER database, has effectively constructed and validated nomograms that can predict the OS and CSS of patients with bone metastasis from primary liver cancer. These nomograms hold great potential in guiding clinical decision-making, aiding in prognosis assessment, and optimizing individualized patient management. Despite the study limitations, our findings contribute to expanding pool of evidence and provide a basis for future advancements in prognostic prediction for PLC patients with bone metastasis.

## Ethics statement

The data was derived from the SEER database, it was unnecessary to obtain patient consent again.

## Consent for publication

Not applicable.

## Funding statement

The work was supported by the 10.13039/501100001809National Natural Science Foundation of China (No.81874062，82072730)

## Data availability statement

The data utilized in this study were sourced from a publicly accessible database. The SEER data, which formed the basis of our analysis, can be obtained from https://seer.Cancer.gov/. Additionally, the specific dataset referenced in the article is readily available within both the main article and supplementary material.

## CRediT authorship contribution statement

**Peng Qiu:** Writing – review & editing, Writing – original draft. **Yunxiang Feng:** Visualization, Supervision. **Kai Zhao:** Supervision, Data curation. **Yuanxin Shi:** Supervision, Conceptualization. **Xiangyu Li:** Supervision. **Zhengdong Deng:** Supervision. **Jianming Wang:** Writing – review & editing, Supervision, Funding acquisition.

## Declaration of competing interest

The authors declare that they have no known competing financial interests or personal relationships that could have appeared to influence the work reported in this paper.
