# Supplementary material for: Predictive models and treatment efficacy for liver cancer patients with bone metastases: A comprehensive analysis of prognostic factors and nomogram development
Source: Heliyon. 2024 Sep 19;10(19):e38038. doi: 10.1016/j.heliyon.2024.e38038 (PMC11462488; doi:10.1016/j.heliyon.2024.e38038)
Supplement: Multimedia component 1 [file mmc1.docx]

**Table S1** Patient characteristics comparison before and after propensity score matching (PSM) among individuals receiving surgical treatment.

| Characteristics | Surgery before PSM | | | Surgery after PSM | | |
| --- | --- | --- | --- | --- | --- | --- |
|  | No | Yes | P value | No | Yes | P value |
| n | 446 | 24 |  | 24 | 24 |  |
| Age, n (%) |  |  | 0.187 |  |  | 1 |
| <=60 | 130 (27.7%) | 4 (0.9%) |  | 3 (6.2%) | 4 (8.3%) |  |
| >60 | 316 (67.2%) | 20 (4.3%) |  | 21 (43.8%) | 20 (41.7%) |  |
| Race, n (%) |  |  | 0.959 |  |  | 0.918 |
| White | 316 (67.2%) | 17 (3.6%) |  | 18 (37.5%) | 17 (35.4%) |  |
| Black | 63 (13.4%) | 3 (0.6%) |  | 3 (6.2%) | 3 (6.2%) |  |
| Other | 67 (14.3%) | 4 (0.9%) |  | 3 (6.2%) | 4 (8.3%) |  |
| Marital status, n (%) |  |  | 0.483 |  |  | 0.22 |
| Married | 255 (54.3%) | 11 (2.3%) |  | 7 (14.6%) | 11 (22.9%) |  |
| Single | 86 (18.3%) | 5 (1.1%) |  | 3 (6.2%) | 5 (10.4%) |  |
| Other | 105 (22.3%) | 8 (1.7%) |  | 14 (29.2%) | 8 (16.7%) |  |
| Sex, n (%) |  |  | 0.01 |  |  | 0.771 |
| Male | 365 (77.7%) | 14 (3%) |  | 13 (27.1%) | 14 (29.2%) |  |
| Female | 81 (17.2%) | 10 (2.1%) |  | 11 (22.9%) | 10 (20.8%) |  |
| Grade, n (%) |  |  | 0.415 |  |  | 0.91 |
| Well differentiated;  Grade I | 97 (20.6%) | 6 (1.3%) |  | 5 (10.4%) | 6 (12.5%) |  |
| Moderately differentiated; Grade II | 176 (37.4%) | 8 (1.7%) |  | 9 (18.8%) | 8 (16.7%) |  |
| Poorly differentiated; Grade III | 168 (35.7%) | 9 (1.9%) |  | 8 (16.7%) | 9 (18.8%) |  |
| Undifferentiated;  Grade IV | 5 (1.1%) | 1 (0.2%) |  | 2 (4.2%) | 1 (2.1%) |  |
| Histological type, n (%) |  |  | 0.149 |  |  | 1 |
| HCC | 332 (70.6%) | 21 (4.5%) |  | 22 (45.8%) | 21 (43.8%) |  |
| CCA | 114 (24.3%) | 3 (0.6%) |  | 2 (4.2%) | 3 (6.2%) |  |
| AJCC T stage, n (%) |  |  | 0.487 |  |  | 0.811 |
| T1 | 137 (29.1%) | 10 (2.1%) |  | 12 (25%) | 10 (20.8%) |  |
| T2 | 102 (21.7%) | 5 (1.1%) |  | 3 (6.2%) | 5 (10.4%) |  |
| T3 | 170 (36.2%) | 6 (1.3%) |  | 7 (14.6%) | 6 (12.5%) |  |
| T4 | 37 (7.9%) | 3 (0.6%) |  | 2 (4.2%) | 3 (6.2%) |  |
| AJCC N stage, n (%) |  |  | 0.167 |  |  | 1 |
| N0 | 313 (66.6%) | 20 (4.3%) |  | 19 (39.6%) | 20 (41.7%) |  |
| N1 | 133 (28.3%) | 4 (0.9%) |  | 5 (10.4%) | 4 (8.3%) |  |
| Tumor size, median (IQR) | 79 (55, 112) | 69 (46.75, 110.5) | 0.528 | 82.208 ± 47.676 | 82.708 ± 50.746 | 0.972 |
| Brain metastasis, n (%) |  |  | 0.596 |  |  | 1 |
| No | 430 (91.5%) | 23 (4.9%) |  | 23 (47.9%) | 23 (47.9%) |  |
| Yes | 16 (3.4%) | 1 (0.2%) |  | 1 (2.1%) | 1 (2.1%) |  |
| Lung metastasis, n (%) |  |  | 0.317 |  |  | 0.477 |
| No | 331 (70.4%) | 20 (4.3%) |  | 18 (37.5%) | 20 (41.7%) |  |
| Yes | 115 (24.5%) | 4 (0.9%) |  | 6 (12.5%) | 4 (8.3%) |  |
| Radiotherapy, n (%) |  |  | 0.338 |  |  | 0.755 |
| No | 253 (53.8%) | 16 (3.4%) |  | 17 (35.4%) | 16 (33.3%) |  |
| Yes | 193 (41.1%) | 8 (1.7%) |  | 7 (14.6%) | 8 (16.7%) |  |
| Chemotherapy, n (%) |  |  | 0.229 |  |  | 1 |
| No | 186 (39.6%) | 13 (2.8%) |  | 13 (27.1%) | 13 (27.1%) |  |
| Yes | 260 (55.3%) | 11 (2.3%) |  | 11 (22.9%) | 11 (22.9%) |  |
