# Supplementary material for: Predictive models and treatment efficacy for liver cancer patients with bone metastases: A comprehensive analysis of prognostic factors and nomogram development
Source: Heliyon. 2024 Sep 19;10(19):e38038. doi: 10.1016/j.heliyon.2024.e38038 (PMC11462488; doi:10.1016/j.heliyon.2024.e38038)
Supplement: Multimedia component 2 [file mmc2.docx]

**Table S2** Patient characteristics comparison before and after propensity score matching (PSM) among individuals receiving radiotherapy.

| Characteristics | Radiotherapy before PSM | | | Radiotherapy after PSM | | |
| --- | --- | --- | --- | --- | --- | --- |
|  | No | Yes | P value | No | Yes | P value |
| n | 269 | 201 |  | 150 | 150 |  |
| Age, n (%) |  |  | 0.374 |  |  |  |
| <=60 | 81 (17.2%) | 53 (11.3%) |  | 41 (13.7%) | 41 (13.7%) | 0.503 |
| >60 | 188 (40%) | 148 (31.5%) |  | 109 (36.3%) | 109 (36.3%) |  |
| Race, n (%) |  |  | 0.375 |  |  |  |
| White | 184 (39.1%) | 149 (31.7%) |  | 114 (38%) | 105 (35%) |  |
| Black | 42 (8.9%) | 24 (5.1%) |  | 15 (5%) | 19 (6.3%) | 0.96 |
| Other | 43 (9.1%) | 28 (6%) |  | 21 (7%) | 26 (8.7%) |  |
| Marital status, n (%) |  |  | 0.872 |  |  |  |
| Married | 150 (31.9%) | 116 (24.7%) |  | 89 (29.7%) | 90 (30%) |  |
| single | 52 (11.1%) | 39 (8.3%) |  | 24 (8%) | 25 (8.3%) | 0.564 |
| Other | 67 (14.3%) | 46 (9.8%) |  | 37 (12.3%) | 35 (11.7%) |  |
| Sex, n (%) |  |  | **0.035** |  |  |  |
| Male | 208 (44.3%) | 171 (36.4%) |  | 118 (39.3%) | 122 (40.7%) | 0.553 |
| Female | 61 (13%) | 30 (6.4%) |  | 32 (10.7%) | 28 (9.3%) |  |
| Grade, n (%) |  |  | 0.044 |  |  |  |
| Well differentiated;  Grade I | 49 (10.4%) | 54 (11.5%) |  | 35 (11.7%) | 35 (11.7%) |  |
| Moderately differentiated; Grade II | 102 (21.7%) | 82 (17.4%) |  | 61 (20.3%) | 62 (20.7%) |  |
| Poorly differentiated;  Grade III | 114 (24.3%) | 63 (13.4%) |  | 54 (18%) | 51 (17%) | 0.602 |
| Undifferentiated;  Grade IV | 4 (0.9%) | 2 (0.4%) |  | 0 (0%) | 2 (0.7%) |  |
| Histological type, n (%) |  |  | 0.051 |  |  |  |
| HCC | 193 (41.1%) | 160 (34%) |  | 108 (36%) | 112 (37.3%) | 0.98 |
| CCA | 76 (16.2%) | 41 (8.7%) |  | 42 (14%) | 38 (12.7%) |  |
| AJCC T stage, n (%) |  |  | 0.854 |  |  |  |
| T1 | 81 (17.2%) | 66 (14%) |  | 45 (15%) | 47 (15.7%) |  |
| T2 | 61 (13%) | 46 (9.8%) |  | 36 (12%) | 33 (11%) |  |
| T3 | 105 (22.3%) | 71 (15.1%) |  | 56 (18.7%) | 57 (19%) | 0.897 |
| T4 | 22 (4.7%) | 18 (3.8%) |  | 13 (4.3%) | 13 (4.3%) |  |
| AJCC N stage, n (%) |  |  | **0.001** |  |  |  |
| N0 | 175 (37.2%) | 158 (33.6%) |  | 110 (36.7%) | 109 (36.3%) | 0.637 |
| N1 | 94 (20%) | 43 (9.1%) |  | 40 (13.3%) | 41 (13.7%) | 1 |
| Tumor size, median (IQR) | 83 (58, 120) | 75 (52, 103) | **0.016** | 78 (55.25, 120.75) | 79 (55, 105) |  |
| Brain metastasis, n (%) |  |  | 0.173 |  |  |  |
| No | 262 (55.7%) | 191 (40.6%) |  | 144 (48%) | 144 (48%) | 0.564 |
| Yes | 7 (1.5%) | 10 (2.1%) |  | 6 (2%) | 6 (2%) |  |
| Lung metastasis, n (%) |  |  | **0.001** |  |  |  |
| No | 186 (39.6%) | 165 (35.1%) |  | 122 (40.7%) | 118 (39.3%) | 0.607 |
| Yes | 83 (17.7%) | 36 (7.7%) |  | 28 (9.3%) | 32 (10.7%) |  |
| Surgery, n (%) |  |  | 0.338 |  |  |  |
| No | 253 (53.8%) | 193 (41.1%) |  | 141 (47%) | 143 (47.7%) | 0.813 |
| Yes | 16 (3.4%) | 8 (1.7%) |  | 9 (3%) | 7 (2.3%) |  |
| Chemotherapy, n (%) |  |  | **< 0.001** |  |  |  |
| No | 137 (29.1%) | 62 (13.2%) |  | 60 (20%) | 58 (19.3%) |  |
| Yes | 132 (28.1%) | 139 (29.6%) |  | 90 (30%) | 92 (30.7%) |  |
