# Supplementary material for: Predictive models and treatment efficacy for liver cancer patients with bone metastases: A comprehensive analysis of prognostic factors and nomogram development
Source: Heliyon. 2024 Sep 19;10(19):e38038. doi: 10.1016/j.heliyon.2024.e38038 (PMC11462488; doi:10.1016/j.heliyon.2024.e38038)
Supplement: Multimedia component 3 [file mmc3.docx]

**Table S3** Patient characteristics comparison before and after propensity score matching (PSM) among individuals receiving chemotherapy.

| Characteristics | | Chemotherapy before PSM | | | Chemotherapy after PSM | | |
| --- | --- | --- | --- | --- | --- | --- | --- |
|  |  | No | Yes | P value | No | Yes | P value |
| n | | 199 | 271 |  | 141 | 141 |  |
| Age, n (%) | |  |  | **0.026** |  |  | 0.345 |
| <=60 | | 46 (9.8%) | 88 (18.7%) |  | 41 (14.5%) | 34 (12.1%) |  |
| >60 | | 153 (32.6%) | 183 (38.9%) |  | 100 (35.5%) | 107 (37.9%) |  |
| Race, n (%) | |  |  | 0.867 |  |  | 0.888 |
| White | | 143 (30.4%) | 190 (40.4%) |  | 98 (34.8%) | 96 (34%) |  |
| Black | | 26 (5.5%) | 40 (8.5%) |  | 21 (7.4%) | 20 (7.1%) |  |
| Other | | 30 (6.4%) | 41 (8.7%) |  | 22 (7.8%) | 25 (8.9%) |  |
| Marital status, n (%) | |  |  | **0.002** |  |  | 0.773 |
| Married | | 94 (20%) | 172 (36.6%) |  | 71 (25.2%) | 77 (27.3%) |  |
| single | | 45 (9.6%) | 46 (9.8%) |  | 32 (11.3%) | 29 (10.3%) |  |
| Other | | 60 (12.8%) | 53 (11.3%) |  | 38 (13.5%) | 35 (12.4%) |  |
| Sex, n (%) | |  |  | 0.912 |  |  | 1 |
| Male | | 160 (34%) | 219 (46.6%) |  | 112 (39.7%) | 112 (39.7%) |  |
| Female | | 39 (8.3%) | 52 (11.1%) |  | 29 (10.3%) | 29 (10.3%) |  |
| Grade, n (%) | |  |  | 0.681 |  |  | 0.97 |
| Well differentiated; Grade I | | 41 (8.7%) | 62 (13.2%) |  | 30 (10.6%) | 29 (10.3%) |  |
| Moderately differentiated; Grade II | | 75 (16.0%) | 109 (23.2%) |  | 53 (18.8%) | 57 (20.2%) |  |
| Poorly differentiated;  Grade III | | 81 (17.2%) | 96 (20.4%) |  | 56 (19.9%) | 53 (18.8%) |  |
| Undifferentiated;  Grade IV | | 2 (0.4%) | 4 (0.9%) |  | 2 (0.7%) | 2 (0.7%) |  |
| Histological type, n (%) | |  |  | **< 0.001** |  |  | 1 |
| HCC | | 171 (36.4%) | 182 (38.7%) |  | 113 (40.1%) | 113 (40.1%) |  |
| CCA | | 28 (6.0%) | 89 (18.9%) |  | 28 (9.9%) | 28 (9.9%) |  |
| AJCC T stage, n (%) | |  |  | 0.534 |  |  | 0.401 |
| T1 | | 64 (13.6%) | 83 (17.7%) |  | 42 (14.9%) | 47 (16.7%) |  |
| T2 | | 39 (8.3%) | 68 (14.5%) |  | 32 (11.3%) | 23 (8.2%) |  |
| T3 | | 77 (16.4%) | 99 (21.1%) |  | 52 (18.4%) | 60 (21.3%) |  |
| T4 | | 19 (4.0%) | 21 (4.5%) |  | 15 (5.3%) | 11 (3.9%) |  |
| AJCC N stage, n (%) | |  |  | 0.15 |  |  | 0.681 |
| N0 | | 148 (31.5%) | 185 (39.4%) |  | 104 (36.9%) | 107 (37.9%) |  |
| N1 | | 51 (10.9%) | 86 (18.3%) |  | 37 (13.1%) | 34 (12.1%) |  |
| Tumor size, median (IQR) | 76 (51.5, 107) | | 81 (55, 113) | 0.211 | 76 (50, 105) | 74 (55, 110) | 0.898 |
| Brain metastasis, n (%) | |  |  | 0.368 |  |  | 0.758 |
| No | | 190 (40.4%) | 263 (56%) |  | 135 (47.9%) | 136 (48.2%) |  |
| Yes | | 9 (1.9%) | 8 (1.7%) |  | 6 (2.1%) | 5 (1.8%) |  |
| Lung metastasis, n (%) | |  |  | 0.575 |  |  | 0.497 |
| No | | 146 (31.1%) | 205 (43.6%) |  | 102 (36.2%) | 107 (37.9%) |  |
| Yes | | 53 (11.3%) | 66 (14%) |  | 39 (13.8%) | 34 (12.1%) |  |
| Surgery, n (%) | |  |  | 0.229 |  |  | 1 |
| No | | 186 (39.6%) | 260 (55.3%) |  | 133 (47.2%) | 133 (47.2%) |  |
| Yes | | 13 (2.8%) | 11 (2.3%) |  | 8 (2.8%) | 8 (2.8%) |  |
| Radiotherapy, n (%) | |  |  | **< 0.001** |  |  | 0.904 |
| No | | 137 (29.1%) | 132 (28.1%) |  | 83 (29.4%) | 84 (29.8%) |  |
| Yes | | 62 (13.2%) | 139 (29.6%) |  | 58 (20.6%) | 57 (20.2%) |  |
